# Workshop report: Identifying opportunities for global integration of toxicogenomics databases, 26–27 June 2013, Research Triangle Park, NC, USA

**DOI:** 10.1007/s00204-014-1387-3

**Published:** 2014-10-19

**Authors:** Diana M. Hendrickx, Rebecca R. Boyles, Jos C. S. Kleinjans, Allen Dearry

**Affiliations:** 1 Department of Toxicogenomics, Maastricht University, Universiteitssingel 40, 6229 ER Maastricht, The Netherlands; 2 P.O. Box 616, 6200 MD Maastricht, The Netherlands; 3 Department of Health and Human Services, National Institute of Environmental Health Sciences, National Institutes of Health, 111 T.W. Alexander Drive, Research Triangle Park, NC 27709 USA

**Keywords:** Databases, Data sharing, Hazard identification, Human, Omics technologies, Risk assessment

## Abstract

A joint US-EU workshop on enhancing data sharing and exchange in toxicogenomics was held at the National Institute for Environmental Health Sciences. Currently, efficient reuse of data is hampered by problems related to public data availability, data quality, database interoperability (the ability to exchange information), standardization and sustainability. At the workshop, experts from universities and research institutes presented databases, studies, organizations and tools that attempt to deal with these problems. Furthermore, a case study showing that combining toxicogenomics data from multiple resources leads to more accurate predictions in risk assessment was presented. All participants agreed that there is a need for a web portal describing the diverse, heterogeneous data resources relevant for toxicogenomics research. Furthermore, there was agreement that linking more data resources would improve toxicogenomics data analysis. To outline a roadmap to enhance interoperability between data resources, the participants recommend collecting user stories from the toxicogenomics research community on barriers in data sharing and exchange currently hampering answering to certain research questions. These user stories may guide the prioritization of steps to be taken for enhancing integration of toxicogenomics databases.

## Background

Traditional approaches for the assessment of toxicological properties of compounds rely heavily on animal testing (Chen et al. 2012). Several issues related to animal experiments have led to the need for alternative experimental methods. Ethical issues concerning animal welfare have resulted in new legislation limiting animal testing, e.g. the Directive on Cosmetic Products (European Commission 2010b). Registration, Evaluation, Authorization and restriction of Chemicals (REACH; European Commission 2007) provides recommendations to limit animal experiments. Furthermore, studies have shown that the results of multiple animal models for evaluating chemical safety are not reliably applicable to humans and vice versa (Hartung 2009).

The consequent need for alternatives to animal testing has led to the expansion of toxicogenomics, e.g. a subfield of toxicology that applies—omics technologies for risk assessment, as a part of the broader application of ‘omics’ technologies for testing toxicity (Sycheva et al. 2013).

The report ‘Toxicity Testing in the 21th Century: A Vision and Strategy’ of the National Research Council (NRC) in the USA (National Research Council (NRC) 2007) states that understanding adverse outcomes of compounds requires a large amount of data, which have to be made publicly available. However, to date, existing public databases for toxicogenomics and other toxicity data only exist to a small extent compared to what is envisioned in the report. Here, several issues are of concern:

- Many toxicogenomics and other toxicity data have been generated, of which large amounts are now stored in separate databases (or not at all, for that matter). However, problems related to public data availability, data quality, interoperability (the ability to exchange information), standardization and sustainability hamper efficient use of these data.
- Reasons for not making data publicly available are related to confidentiality, personal privacy and intellectual property (Briggs et al. 2012). Furthermore, not all funding agencies and journals require that data are made publicly available (Woodall and Goldberg 2008). There is a lack of support for data collection and deposition, and facilities for local data storage in individual laboratories have to be increased (Waters and Yauk 2007). Moreover, data are pre-processed and normalized following a specific protocol from a particular laboratory, instead of having standardized pre-processing and normalization workflows (Woodall and Goldberg 2008). Furthermore, complex datasets are often fragmented across different databases, because databases are often limited to only capturing specific data types (Sansone et al. 2012).
- It has been observed that some datasets in public databases are of low quality due to insufficient data curation (Waters and Yauk 2007). Moreover, experimental design and protocols are often poorly explained (Benfenati et al. 2010; Christensen et al. 2011; Waters and Yauk 2007). Another common issue is that data are often not well organized, so that they can be difficult to find (Berg et al. 2011).
- In addition, datasets are frequently deposited in separate databases in different formats and with different annotations (Sansone et al. 2012), which hampers interoperability between databases. Furthermore, there are insufficient bioinformatics tools to integrate the various data (Berg et al. 2011; Briggs et al. 2012).
- Overall, there is a lack of standardization among different databases because each field uses its own agreements (Benfenati et al. 2010; Sansone et al. 2012). Further, disparate laboratories have their own experimental protocols, preprocessing and normalization procedures (Berg et al. 2011), which makes it difficult to compare and combine data from different laboratories.
- Problems with creating a sustainable data infrastructure are complicated by the lack of available funding in proportion to the increase in amount and complexity of data (Christensen et al. 2011; Waters and Yauk 2007). Moreover, people are often not aware of the existence of current databases and their optimal usage, due to insufficient communication, dissemination, education and training (Berg et al. 2011; Waters and Yauk 2007).

To discuss these and other challenges in toxicogenomics data management, a joint EU-US workshop was organized, sponsored by Research Data Alliance (RDA) Europe and the National Institute of Environmental Health Sciences (NIEHS; NC, USA), which focused on the identification of opportunities for global integration of toxicogenomics databases. Contributions from universities and research institutes helped to get insight in current data sharing and exchange in toxicogenomics. Gaps and needs were identified, and opportunities for improvement were discussed. This report summarizes the workshop presentations, discussions and resulting recommendations.

## Workshop presentations and discussion

Currently, there are increasing demands on chemical risk assessment (Jos Kleinjans, Maastricht University). Because of the high failure rate of new drug candidates due to unmanageable toxicity, there is a need for improved approaches for human risk assessment. Further, the EU REACH programme on industrial chemicals requires that existing and new substances should be subject to the same toxicity testing procedure under a single system. At present, thousands of industrial chemicals are placed on the market without proper safety dossiers. As a consequence, large amounts of additional tests are required. Additionally, in light of the EU-wide ban on animal use in cosmetics development [Council Directive 92/32/EEC (7th Amendment)], there is a need for alternative toxicity tests that are not based on animal experimentation. Other EU regulations, e.g. on food chemicals, are directed towards reducing animal testing.

In the amendment to the latest consolidated version of the REACH legislation, approaches based on toxicogenomics are suggested as alternatives to animal testing. Toxicity testing based on high-throughput ‘omics’ data would involve inference of toxicity pathways from large amounts of data [National Research Council (NRC) 2007]. In this context, several reports provide recommendations on the storage and use of big data in toxicogenomics. The NRC report “Toxicity Testing in the 21th Century” [National Research Council (NRC) 2007] mentions the need for central repositories for—omics data. In “A digital agenda for Europe” (European Commission 2010a), the European commission points out that the scientific community has to make use of existing data as much as possible, instead of reproducing data that have already been generated before. Against this background, the Blue Ribbon Task Force on Sustainable Digital Preservation and Access indicates that there is a risk that data get lost and that preservation actions need to be taken to assure that data will be available in the future (Blue Ribbon Task Force on Sustainable Digital Preservation and Access 2010).

The amount of toxicity data is expected to increase in the future. Microarray, proteomics, metabolomics and epigenomics require several megabytes of storage per experiment. Next-generation sequencing (NGS) will require several terabytes of storage per experiment. Furthermore, the application of chemoinformatics is expanding, yielding an increasing number of data resources. For instance, eChemPortal (http://www.echemportal.org) already captures more than 25 databases on chemical, physical and toxicity information.

Data are driving solutions to complex science and societal challenges (Beth Plale, Indiana University). Sharing and exchange of data will lead to an acceleration of innovation. However, integrating data requires infrastructure development, including common metadata standards, an integration framework, data access and preservation policy and practice, harmonized standards, a common economic model for sustaining data, digital object identifiers and tools for data discoverability. A number of organizations are addressing this need. One of these is the Research Data Alliance (RDA) (https://rd-alliance.org/), a global community-driven organization launched in March 2013 to accelerate data-driven innovation. The focus is on building the infrastructure necessary to enhance data sharing and exchange. Furthermore, the RDA aims to accelerate the development of a coordinated global data infrastructure. The RDA operates through its interest and working groups. RDA interest groups focus on the infrastructure needed for data sharing and exchange. RDA working groups focus on short-term deliverables enhancing data sharing and exchange, which can be achieved in a period of 12–18 months.

The RDA Europe project (https://europe.rd-alliance.org/), the European plug-in of RDA, focuses on coordinating a series of cross-infrastructure experiments, called prototypes, on global interoperability with a selected group of projects and communities. Each prototype identifies solutions and the remaining challenges for enhancing data sharing and exchange in a particular field of research. One prototype focuses on global connections between toxicogenomics databases and other toxicity data resources, with the main focus on connecting EU and US databases. The workshop was organized in the framework of RDA Europe, in collaboration with NIEHS, in order to discuss available EU and US data resources and to identify gaps and needs that hamper interoperability.

### Toxicogenomics data repositories accepting data submissions

#### *Chemical Effects in Biological Systems (CEBS)* (Jennifer Fostel, NIEHS)

The Chemical Effects in Biological Systems (CEBS) (http://cebs.niehs.nih.gov) is a curated, relational, public database of data sets from universities, industry and governmental institutes developed by the National Toxicology Program (NTP). The database houses data from a number of different experiment types, including animal studies, cellular assays [histopathology, clinical pathology, genetic toxicology, immunotoxicology, polymerase chain reaction (PCR)] and microarray data. Metadata describe the biological context, study design, time line, protocols and participant characteristics. Conclusions like trial/trend results, fold changes and activity calls are also deposited in the database. Data from CEBS can be queried and integrated across studies. Furthermore, CEBS provides tools for visual data mining of all data in a domain (an assay or a chemical).

Data are uploaded into the database into a unified format: the SIFT (Simple Investigation Formatted Text) format (Waters et al. 2008). The syntax of SIFT is based on the SOFT (Simple Omnibus Format in Text) format used in the Gene Expression Omnibus (GEO) database (www.ncbi.nlm.nih.gov/geo/). The SIFT format is used to capture study metadata, study data and data transformation. SIFT uses Microarray Gene Expression-Tabular (MAGE-TAB) for microarray data, a format compliant with the Minimal Information About a Microarray Experiment (MIAME). Scientists willing to deposit their data into CEBS can create a SIFT file incorporating their data and metadata using the CEBS DataBuilder, a java tool that can be downloaded from the CEBS website. The CEBS data dictionary (CEBS-DD) is a compendium of toxicological terms and their synonyms used in a variety of toxicological sources. Several repositories for toxicogenomics and other toxicity data, exchange formats for toxicity data and Lab Information Management Systems (LIMS) were studied in order to ensure a comprehensive data dictionary that is consistent with other efforts (Fostel et al. 2005). The CEBS data dictionary was originally text based, but is now in Extensible Markup Language (XML) format. Currently, the CEBS data dictionary is aligned with Standards for Exchange of Nonclinical Data (SEND, http://www.cdisc.org/send) and Ontology for Biomedical Investigations (OBI, http://bioportal.bioontology.org/ontologies/OBI). When scientists deposit data into CEBS, curators verify manually that the studies contain biologically sensible information. Furthermore, individuals depositing data into CEBS have to confirm that the study is correctly presented. When importing data from other sources, automated scripts are used to check whether the studies are imported correctly. CEBS works with pathologists and ontologists on the consolidation of ontology terms. Currently, CEBS is working on a project toward linked data (structured and computer-readable data that can be interlinked).

#### *The diXa data infrastructure* (Ugis Sarkans, EBML-EBI)

The diXa infrastructure (http://www.dixa-fp7.eu/) consists of a central data warehouse containing data from toxicogenomics project repositories and public databases. Apart from data from European projects, diXa also includes the public data from the National Project of Toxicogenomics in Japan, named Open TG-GATES (Toxicogenomics Project-Genomics Assisted Evaluation System, see below for more details).

The diXa data warehouse connects multi-omics studies, deposited in EBI resources [ArrayExpress for transcriptomics (http://www.ebi.ac.uk/arrayexpress/), PRIDE for proteomics (http://www.ebi.ac.uk/pride/archive/), MetaboLights for metabolomics (http://www.ebi.ac.uk/metabolights/)] and provides a single interface for search and retrieval of “omics” data. In this way, data can be reused in cross-platform projects.

The diXa data warehouse is connected to a chemicals database portal and a human diseases database portal. Data from external sources are integrated in the chemicals database portal via InChI/Keys (textual identifiers for chemical substances). The chemicals database portal provides cross-links to other databases with chemicals and their activities. In the human diseases database portal, molecular data on human diseases are retrieved from public data sources.

All data submitted to the diXa warehouse are subjected to quality control using Genedata Expressionist^®^ (https://www.genedata.com/products/expressionist/) quality control pipelines. When a data set is submitted to diXa, the diXa staff checks whether the data and metadata are in an acceptable format. They also examine whether the metadata are complete and that the data can correctly be read in the specified format. Furthermore, the diXa staff double-checks with submitters that the information accurately reflects the experimental procedures performed. Moreover, the data have to pass a minimum of quality control procedures. Finally, requirements that have to be fulfilled so that data can be properly analysed (e.g. control samples for comparisons, enough samples) are checked.

Data and metadata have to be uploaded in ISA-TAB format (Rocca-Serra et al. 2010). The ISA-TAB format provides fields for the metadata and links to the external files containing the data.

To keep researchers up to date on the development of the diXa infrastructure, training courses and workshops are organized on a regular basis at different locations in Europe. Stakeholders are engaged in the diXa project by conducting a survey of other EU toxicogenomics projects, in order to understand data and user expectations and to build trust.

### Toxicogenomics databases primarily intended as information resources

#### *Comparative Toxicogenomics Database (CTD)* (Caroline Mattingly, North Carolina State University)

Currently, more than 60,000 toxic substances are on the market, and ~2,000 substances are added each year. Approximately 8,000 chemicals are carcinogens. For ~40 % of the 3,300 chemicals that are produced in high amounts, no toxicity data are available. Moreover, full toxicity data are available for only 25 % of the chemicals that are found in consumer products. This hampers obtaining insight in how chemicals affect our health. The Comparative Toxicogenomics Database (CTD) (http://ctdbase.org/) is a manually curated repository of chemicals, genes, diseases and their interactions from the curated scientific literature, which has the aim to improve the understanding of the adverse effects of chemicals on our health. CTD provides tools for visualizing the interactions and to perform enrichment analysis on a set of genes or chemicals.

CTD includes over 12,000 chemicals, over 30,000 genes and over 6,000 diseases. Furthermore, CTD contains more than 860,000 curated chemical–gene interactions, more than 27,000 curated gene–disease relationships and more than 180,000 curated chemical–disease relationships. Over 300 manuscripts use CTD data, and more than 30 public databases incorporate CTD data.

CTD uses several ontologies, including EntrezGeneID, Medical Subject Headings (MeSH; Davis et al. 2009) and CTD’s merged disease vocabulary (MEDIC; Davis et al. 2012). Gene ontology (GO) and pathways from KEGG (Kyoto Encyclopedia of Genes and Genomes) and Reactome are integrated into CTD (Davis et al. 2013). CTD is also developing exposure ontology (ExO) and will integrate exposure and phenotypic data with the other information in the database in order to infer relationships between chemicals, phenotypes and diseases (Mattingly et al. 2012).

#### *Library of Integrated Network Based Cellular Signatures (LINCS)* (Aravind Subramanian, Broad Institute)

The Library of Integrated Network Based Cellular Signatures (LINCS) (http://www.lincsproject.org/) aims to serve as a source for improving the understanding of cellular activity and is based on Connectivity Map (CMap). CMap (https://www.broadinstitute.org/cmap/) is a database that uses mRNA expression signatures from cultured human cells treated with bioactive small molecules to connect diseases, genes and drugs. The database includes 453 Affymetrix profiles for 164 drugs. CMap has more than 16,000 users and is cited in more than 900 publications (Lamb et al. 2006). LINCS expands CMap by including more small molecules, genomic perturbations, cellular contexts and treatment parameters. The representation of the human transcriptome is reduced taking advantage of correlation in gene expression. A set of 1,000 landmark genes is selected that captures approximately 80 % of the correlations in gene expression profiles. The complete list of landmark genes is available on http://www.lincscloud.org/l1000/. Currently, LINCS contains expression profiles for 5,178 compounds and 15 cell types for the 1,000 landmark genes and 21,000 inferred genes. Furthermore, LINCS contains data from gene knockdown experiments (3,712 genes). Data quality is checked by calculating Spearman correlation between biological replicate signatures.

#### *Toxicogenomics Genomics*-*Assisted Toxicity Evaluation system: Open TG*-*GATEs* (Florian Caiment, Maastricht University)

Open TG-GATEs (http://toxico.nibio.go.jp/english/index.html) is a public database consisting of in vivo (rat) and in vitro (human and rat) clinical, pathological and expression data for approximately 170 toxic compounds from the National Projects of Toxicogenomics in Japan, generated from liver and kidney. The database contains more than 20,000 Affymetrix microarray profiles. The experimental design is the same for each compound. In vivo (liver and kidney) data consist of three biological replicates, measured at eight time points and four dose levels. The in vitro (rat and human hepatocyte) data consist of two biological replicates, measured at three time points and four dose levels. Microarray profiles are stored as CEL files. Clinical data are displayed as tables, and pathological data as both images and tables.

Data quality is checked by evaluating inter- and intra-laboratory reproducibility of biological replicates by clustering, Spearman correlation and determining the overlap in lists of differentially expressed genes (Noriyuki et al. 2012). Further quality control is performed using the ArrayAnalysis Quality Control pipeline (www.arrayanalysis.org/). Toxygates (http://toxygates.nibio.go.jp/toxygates/) is a platform for analysing the TG-GATEs data (Nystrom-Persson et al. 2013). Given a list of genes or probes, the following tools can be used to analyse the data: KEGG pathway analysis, GO analysis, analysis of ChEMBL or DrugBank targets and compound ranking.

#### *DrugMatrix* (Scott Auerbach, NIEHS)

DrugMatrix (https://ntp.niehs.nih.gov/drugmatrix/index.html) is a public, comprehensive, database of gene expression profiles, pathology assays and pharmacology assays hosted as a resource to researchers by the National Toxicology Program (NTP). DrugMatrix contains data from expression studies for over 600 compounds, including ~4,000 dose time–tissue combinations, ~2 million dosed tissue samples, ~18,000 microarrays, ~127,000 histopathology measurements and ~100,000 haematology and chemistry measurements. Furthermore, more than 800 compounds were profiled across 130 in vitro pharmacology assays. The database also provides links to the drug literature (Fielden and Halbert 2007). Currently, the TG-GATEs data are added to DrugMatrix. All data in DrugMatrix are generated through a standardized experimental protocol utilizing rats or primary rat hepatocytes systematically treated with therapeutic, environmental and industrial chemicals at both toxic and non-toxic doses (Ganter et al. 2006). Unprocessed and normalized Affymetrix microarray data can be downloaded from the FTP site (ftp://anonftp.niehs.nih.gov/drugmatrix). DrugMatrix contains hundreds of drug signatures (biomarkers) for liver, heart, kidney, muscle and primary hepatocytes. The DrugMatrix Toolbox and ToxFX provide tools for data analysis (Fielden and Halbert 2007).

The DrugMatrix Toolbox allows users to upload their data or mine the DrugMatrix data. The following data analysis tools are available: finding similar expression profiles, hierarchical clustering, finding consistently changed genes, differentially expressed genes (DEGs) analysis, pathway analysis and gene ontology (GO) analysis. Furthermore, expression profiles can be visualized on pathways. Expression profiles for more than 50 phenotypes can be scored with drug signatures. Expression patterns for putative biomarker sets can be constructed, and the performance of biomarker sets for detecting phenotypes can be tested. Moreover, the DrugMatrix Toolbox provides tools to mine the literature and to identify enriched literature annotations in groups of expression profiles.

ToxFX (https://ntp.niehs.nih.gov/toxfx/) is a rapid, automated toxicogenomics analysis tool including quality control, scoring of expression profiles with drug signatures, DEGs analysis and pathway analysis. ToxFX generates a detailed toxicogenomics report in five minutes.

### Chemoinformatics databases

#### *ChEMBL* (Anne Hersey, EMBL-EBI)

ChEMBL (https://www.ebi.ac.uk/chembl/) is an open-access database of bioactivity data for drug discovery maintained by the European Bioinformatics Institute (EBI). The database, originally developed by a biotechnology company, consists of data manually extracted from the literature, a subset of data from PubChem (https://pubchem.ncbi.nlm.nih.gov/) and deposited data. Bioactivity data are associated with a biological target and a chemical structure. Compounds are stored in a structure-searchable format. Protein targets are linked to protein sequences in UniProt (http://www.uniprot.org/). ChEMBL is updated regularly with new data. ChEMBL covers about 1,300,000 drug-like molecules, over 10,000,000 activities, over 700,000 assays, about 10,000 targets and links to more than 50,000 documents.

For each compound, compound details, assay details, target details and literature references are provided (Gaulton et al. 2012). Several types of targets are included in ChEMBL: proteins, protein complexes, protein families, nucleic acids, cell lines, tissues, sub-cellular fractions and organisms. ChEMBL contains binding, functional and ADMET (Absorption, Distribution, Metabolism, Excretion and Toxicity) assays.

ChEMBL data can be accessed by downloads or web services that allow to search by compound or target. ChEMBL provides links to other public resources, e.g. compound and protein databases (Warr 2009). Additionally, ChEMBL provides tools and resources for data mining.

Currently, ChEMBL is working on importing data from DailyMed (http://dailymed.nlm.nih.gov/dailymed/about.cfm), a database with medication information.

#### *Unified Chemical Identifier System (UniChem)* (Anne Hersey, EMBL-EBI)

Multiple resources hold compound data, and maintaining the links between all these databases is time-consuming. Furthermore, rules for constructing identifiers are not consistent among databases.

UniChem (Unified Chemical Identifier system) tries to cope with these problems by providing important links between databases that contain compound structures [e.g. DrugBank (http://www.drugbank.ca/), ChEMBL]. InChiKeys are used as a unified chemical identifier. UniChem contains data from 16 sources on more than 31,000,000 structures. UniChem provides web services for identifier searching or source mapping between databases (Chambers et al. 2013).

### Microarray and Sequencing Quality Control (Weida Tong, FDA)

The US Food and Drug Administration’s (FDA) MicroArray Quality Control project (MAQC) (http://www.fda.gov/ScienceResearch/BioinformaticsTools/MicroarrayQualityControlProject/) focuses on microarrays and next-generation sequencing technology in three phases. MAQC I assesses the reliability, repeatability and reproducibility of microarray data (Shi et al. 2006). A total of 137 participants from 51 organizations were involved. MAQC II studies the reliability of microarray-based biomarkers in clinical studies and toxicology (Shi et al. 2010). A total of 202 participants from 97 organizations were involved. MAQC III, also called Sequencing Quality Control (SEQC), assesses the reliability of next-generation sequencing (NGS) experiments and compares them with microarrays (Wang et al. 2014). A total of 180 participants from 73 organizations were involved. During the SEQC study, over 10 terabytes of more than 100 billion reads were generated.

Cross-site and cross-platform reproducibility of NGS data were compared to microarrays and quantitative polymerase chain reaction (qPCR). NGS data were more similar to qPCR data than microarrays. Moreover, the identification of differentially expressed genes (DEGs) was compared between NGS and microarrays. When the treatment response was strong, NGS detected more DEGs than microarrays. When the treatment response was weak, Affymetrix microarrays detected more DEGs than NGS. Further, the strength of the treatment response determined the degree of overlap between microarrays and NGS.

Furthermore, samples were randomly split into a training set and a test set with approximately the same size. Classifiers were developed with the training set, and the reliability of these classifiers was examined with the test set. Performance of the classifiers was compared between NGS and microarray data. No significant differences were found between the two platforms.

Finally, 278 pipelines for analysing NGS data were evaluated in order to make a pipeline selection guide for decision-making.

MAQC and SEQC datasets are publicly available from the MAQC website or from another public data resource (GEO, ArrayTrack or CEBS; Shi et al. 2006, 2010).

### WikiLIMS: A Laboratory Information Management System for Toxicogenomics (Stephen Edwards, EPA)

An adverse outcome pathway (AOP) is a series of key events describing how exposure to an environmental stressor leads to an adverse outcome. At the systems biology level, three types of networks are involved in an AOP: the molecular network, the key event network and the population network (Edwards and Preston 2008). To enable systems’ biology approaches, data management systems need to be capable of handling large amounts of heterogeneous data.

WikiLIMS (http://bioteam.net/tag/wikilims/) is a Laboratory Information Management System (LIMS) for toxicogenomics, developed at the United States Environmental Protection Agency (EPA). The goal of WikiLIMS is to enhance human health risk assessment through data integration within EPA. WikiLIMS is a non-public system that can handle many different data types and is easy to update when more data become available. WikiLIMS currently only handles non-public data within the Office of Research and Development (ORD) at the EPA. WikiLIMS provides links to US EPA public databases (ACToR, DSSTox, ExpoCastDB, ToxCastDB and ToxRefDB, see http://actor.epa.gov/).

### Proof of principle of meta-analysis in toxicogenomics (Florian Caiment, Maastricht University)

To demonstrate the strength of combining data from different resources for improving toxicological risk assessment in humans, within the diXa project, a follow-up work on the integration of human disease data with molecular toxicological data derived from human in vitro models was conducted. The research focused on hepatocellular carcinoma (HCC), which is the fifth most common cancer worldwide and the third most common cause of cancer mortality. HCC accounts for approximately 90 % of primary liver cancers and is induced either by hepatic viral infections or hepatic cirrhosis.

Connectivity Map, data from Open TG-GATEs and publicly available data on liver carcinogens [from the International Agency for Research on Cancer (http://www.iarc.fr/), the US EPA, the NTP and the Carcinogenic Potency Project (http://toxnet.nlm.nih.gov/cpdb/)] were integrated with data derived from human HCC (among others from ArrayExpress) to allow better classification of carcinogenic compounds. The results demonstrated that combining data from different sources increased the accuracy of liver carcinogenicity predictions (Caiment et al. 2014).

## General discussion

### Data sharing and exchange in toxicogenomics: gaps and needs

The general discussion after the presentations focused mainly on non-confidential data that can be made publicly available without coping with privacy issues. Four types of problems hampering data sharing and exchange in toxicogenomics were discussed: communication problems, problems related to the quality of the data, sustainability problems and need for training and support.

Communication problems occur between partners of different disciplines. Researchers involved in toxicogenomics projects are often from different disciplines, including toxicologists, data managers and technological persons. Each discipline developed terminologies, reporting guidelines, data formats and metadata formats that are in agreement with a specific set of needs. As a consequence, it is difficult to define standards in the field of toxicogenomics. The best-case scenario would be that scientists from different disciplines define consensus terminologies, standards and formats for toxicogenomics. If it appears not possible to reach consensus, an alternative may be to make existing terminologies, standards and formats interoperable. This can be accomplished by establishing dictionaries with synonyms (e.g. the CEBS data dictionary) and/or mapping between standards.

Reuse of public data in new projects requires that the users have to be certain that the data are of good quality. Problems related to data quality can consist of mistakes in the data and metadata, insufficient metadata and poor quality of the data themselves (in terms of reliability, reproducibility and repeatability). Therefore, data deposited in public repositories have to be subjected to thorough quality control before making it public. Several public databases already fulfil this requirement, but quality control workflows are not harmonized across different data repositories containing data from the same measurement technology. An evaluation of quality control pipelines for each platform, like the one conducted for NGS data in the SEQC study, can guide harmonization of quality control by identifying best practices.

Data infrastructures have to be sustainable, taking into account the evolution of the field. Because data infrastructures and services are not often cited, their impact is underestimated by funding agencies. This leads to problems regarding the funding for extending the databases according to new developments in the field, as well as maintenance of existing resources. The participants of the workshop recommend defining a persistent identifier for the use of data infrastructures. A persistent identifier would enable better citations of the infrastructures, which hopefully would have a positive effect on available funding. Furthermore, in this way, the number of studies per year using a particular database, and thus a measure of impact, can be better reflected by mining the literature.

Generally, data infrastructures have been insufficiently supported. Beyond the infrastructure itself, people involved in using, developing and maintaining data infrastructures, need appropriate training and support. This includes help with implementation, offering courses on e-infrastructures and organizing forums to ask questions and discuss topics.

Table 1 summarizes how the different databases, studies, organizations and tools presented at this workshop attempt to deal with the problems outlined above.

**Table 1:** Summary of the databases, studies, organizations and tools presented at the workshop and how they deal with problems regarding communication, quality of the data, sustainability and education

Database/study/organization/tool	Deals with				How?
	Communication	Quality	Sustainability	Training and support	
RDA	X			X	Working and interest groups Forum
CEBS	X	X	X		Unified format (SIFT) CEBS data dictionary Curation of data Possibility to deposit new data
diXa	X	X	X	X	InChI/Keys for chemicals Unified format for metadata (ISA-TAB) Quality control pipeline (Genedata) Possibility to deposit new data Training and workshops
CTD	X	X	X		Use of ontologies CTD’s merged disease vocabulary Developing exposome ontology Manually curated Updated regularly
LINCS		X			Reproducibility between replicates checked
TG-GATEs	X	X			Same experimental design for all experiments Data checked on reproducibility between replicates
DrugMatrix & ToxFX	X	X			Standardized experimental protocol ToxFX provides tools for quality control
ChEMBL	X		X		Data in a unified format Regularly updated
UniChem	X				Unified chemical identifier
MAQC & SEQC	X	X			Data deposited in public resources that used standardized formats, e.g. CEBS Assess reliability, repeatability and reproducibility of microarrays and NGS data Comparing different quality control pipelines currently in use for NGS data
WikiLIMS			X		Can handle many different data types/formats Easy to update

### Generating a roadmap to enhance interoperability of existing data infrastructures

With a number of issues brought forth during the course of the workshop, any roadmap towards improvement would be a first step. There was agreement that the roadmap should include the structure of the RDA to further advance a number of key issues.

**Fig. 1:**
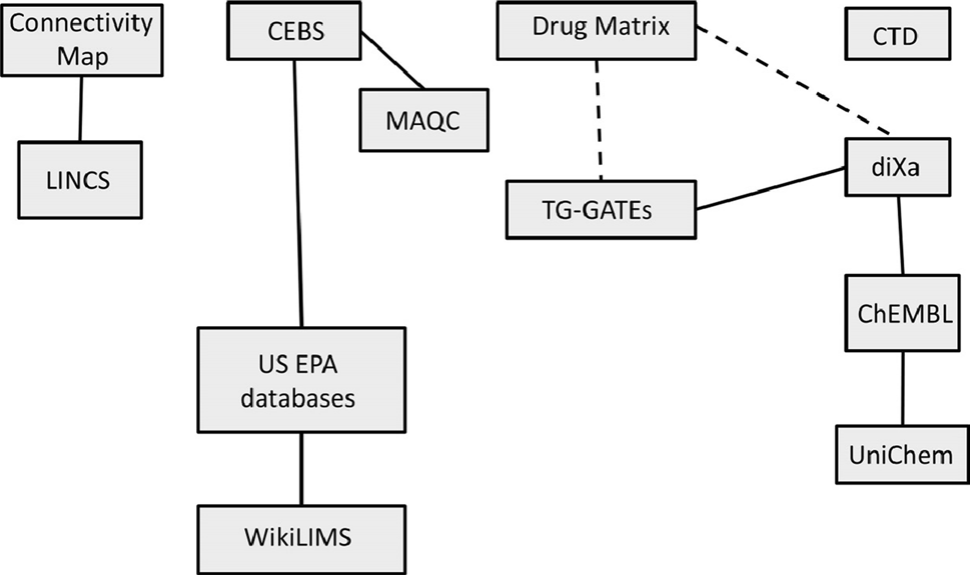
Links between the different data sources presented at the workshops. *Dashed lines* indicate ongoing efforts

The case study presented at the workshop showed that combining data from different sources increased the accuracy of predictions in risk assessment. Some of the data resources presented at the workshop are already linked (see Fig. 1). The workshop participants agreed that providing more links between the different resources will facilitate toxicogenomics research.

Across the existing landscape, resources need to be made easier to find. The workshop participants agreed that there is a need for a web portal that provides links to all databases relevant for toxicogenomics and that gives a description of the information and experimental data that are deposited in each database.

When considering an approach to these issues, it became obvious that any roadmap must be grounded in the needs of potential users. User stories highlighting research currently limited due to a lack of interoperability of data resources would need to be collected. By analysing user stories, more use cases can be defined that are limited by the current scientific gaps due to the lack of data exchange. These use cases can be utilized to support the prioritization of steps that need to be undertaken for linking data resources.
